# In Vitro Antiviral Activities of Salinomycin on Porcine Epidemic Diarrhea Virus

**DOI:** 10.3390/v13040580

**Published:** 2021-03-30

**Authors:** Chen Yuan, Xintong Huang, Ruiyu Zhai, Yichao Ma, Anyuan Xu, Penghao Zhang, Qian Yang

**Affiliations:** MOE Joint International Research Laboratory of Animal Health and Food Safety, College of Veterinary Medicine, Nanjing Agricultural University, Weigang 1, Nanjing 210095, China; yuanchen060624@163.com (C.Y.); hxt17512544263@163.com (X.H.); zhairy2021@163.com (R.Z.); 2020207028@stu.njau.edu.cn (Y.M.); anyuan202103@163.com (A.X.); zphforever@163.com (P.Z.)

**Keywords:** PEDV, salinomycin, MAPK pathway, ROS

## Abstract

Porcine epidemic diarrhea virus (PEDV), an enteropathogenic coronavirus, has catastrophic impacts on the global pig industry. Owing to the lack of effective vaccines and specific therapeutic options for PEDV, it is pertinent to develop new and available antivirals. This study identified, for the first time, a salinomycin that actively inhibited PEDV replication in Vero cells in a dose-dependent manner. Furthermore, salinomycin significantly inhibited PEDV infection by suppressing the entry and post-entry of PEDV in Vero cells. It did not directly interact with or inactivate PEDV particles, but it significantly ameliorated the activation of Erk1/2, JNK and p38MAPK signaling pathways that are associated with PEDV infection. This implied that salinomycin inhibits PEDV replication by altering MAPK pathway activation. Notably, the PEDV induced increase in reactive oxidative species (ROS) was not decreased, indicating that salinomycin suppresses PEDV replication through a pathway that is an independent pathway of viral-induced ROS. Therefore, salinomycin is a potential drug that can be used for treating PEDV infection.

## 1. Introduction

Porcine epidemic diarrhea virus (PEDV) is a porcine enteropathogenic coronavirus whose infections are associated with high morbidity in swine of all ages, as well as a high mortality rate (up to 100%) in suckling piglets, thereby negatively impacting the global pig industry [1,2,3]. Piglets infected with PEDV are characterized by vomiting, watery diarrhea and dehydration [4,5]. In 2010, there was a large-scale outbreak of porcine epidemic diarrhea (PED) in China, while in 2013, the virus also emerged in the United States before rapidly spreading to pose a severe economic and public health risk [2,6]. Following the emergence of the PEDV variant in the United States and China, studies on this virus have increased. Despite the availability of vaccines against PEDV, this virus is still associated with annual epidemics. The available vaccines are not 100% effective [7]. Therefore, antiviral drug therapy has been considered to be a novel and effective way for treating the infections associated with PEDV. Currently, there are no specific treatments for PEDV infection, which necessitates the exploration and development of potential anti-PEDV drugs.

Salinomycin, a monocarboxylic ionophore isolated from *Streptomyces albus*, has been used as an antibiotic [8,9]. Its extensive adoption is due to its high efficacy, spectral properties and positive effects on drug-resistant pathogens. Salinomycin, for example, has been widely used as an anticoccidiosis agent in chickens [10,11]. It has been documented that salinomycin kills cancer stem cells at a 100-fold lower dose than paclitaxel when used to treat mouse breast cancer stem cells [12]. Moreover, the efficacy of salinomycin in controlling Clostridium perfringens Type-A infection in growing pigs has been reported [13]. Although a variety of pharmacological activities associated with salinomycin have been shown, its potential against viral infection has not been elucidated. To our understanding, only one study reported the potential of salinomycin in inhibiting influenza virus infection by disrupting endosomal acidification and viral matrix protein 2 function [13]. Therefore, the antiviral effects of salinomycin on PEDV infections have not been established.

In this study, we aimed at evaluating the antiviral effects and the inhibitory mechanisms of salinomycin against PEDV infection of Vero cells.

## 2. Materials and Methods

### 2.1. Cells, Virus and Reagents

Vero cells were cultured in DMEM supplemented with 10% fetal bovine serum (FBS) and maintained at 37 °C in a humidified 5% CO_2_ incubator. The PEDV live-attenuated strain Zhejiang08 was preserved in our laboratory [14]. Salinomycin was purchased from Selleck Chemicals (Houston, USA), and diluted to 10 mM in DMSO. Antibodies against phospho-p44/42 MAPK (Erk1/2) (Thr202/Tyr204), Erk1/2, phospho-p38MAPK (Thr180/Tyr182), p38MAPK, phospho-JNK((Thr183/Tyr185), JNK, GAPDH and HRP labeled secondary antibodies, including goat anti-mouse IgG and goat anti-rabbit IgG, were sourced from Cell Signaling Technology (Beverly, MA, USA).

### 2.2. Cell Viability Assay

Cytotoxic effects of salinomycin on Vero cells were detected through Cell Counting Kit-8 (CCK8, beyotime, Shanghai, China) assay. Vero cells were cultured in 96-well plates for 12 h. The salinomycin-treated cell suspension (100 μL/ well) was inoculated into 96-well plates following its pre-incubation in a humidified incubator (37 °C, 5% CO_2_) for 24 h. Then, 10 μL of the CCK-8 solution was added into each well of the plate and further cultured for 4 h. Absorbance was measured at 450 nm using a microplate reader. Cell viability (%) was determined as the percentage ratio of absorbance of the samples versus the untreated control.

### 2.3. Virus Replication Inhibition Assay

Serum starved Vero cells (5 × 10^5^) in 24-well plates were pretreated with salinomycin at various concentrations (0.01, 0.1 and 1 μm) for 1 h at 37 °C, and then infected with PEDV at an MOI of 0.1 for 1 h. Then cells were treated with a corresponding inhibitor, washed extensively using PBS and supplemented with DMEM (with or without inhibitors) in each well. At 24 hpi, the viral yield was titrated in Vero cells and the results expressed by TCID50/mLor plaque assays.

To test whether salinomycin (1 μM) affected the viral entry or post entry stages of PEDV infection, serum starved Vero cells were incubated with PEDV for 1 h at 4 °C to allow the viruses to adsorb to the cells membrane but not to penetrate the cells. The cells were washed with ice-cold PBS. To identify the effect of salinomycin (1 μM) on the virus entry process, pre-warmed fresh medium was supplemented with DMSO or salinomycin (1 μM) after which the cell cultures were quickly maintained at 37 °C for 1 h to allow viruses entry into the Vero cells. The purpose was to determine the possibility that salinomycin could affect the viral entry stages of PEDV infection. Thereafter, fresh medium was replaced and re-incubated for 24 h at 37 °C. Simultaneously, to identify the effect of salinomycin (1 μM) on the PEDV post entry process, the PEDV adsorbed Vero cells were placed at 37 °C for 1 h to allow the virus to entry into the Vero cells. Then, it was incubated for 24 h at 37 °C with or without salinomycin. The viral yields were titrated in Vero cells and the results were expressed by TCID50/mL.

In testing the potential of salinomycin to affect the virus binding to Vero cells, serum-starved Vero cells in 6-well plates were incubated in the absence or presence of salinomycin at a concentration of 1 μM at 37 °C for 1 h. This was followed by incubation with virus stock at 4 °C for 1 h allowing the infusion of viruses into the cell membrane, in the absence or presence of salinomycin. Thereafter, cells were subjected to two rounds of freezing-thawing to release cell membrane attached to viruses. After centrifugation, the virus yield in Vero cells was titrated by TCID50 assay.

### 2.4. The Plaque Forming Assay

The viral titer of supernatant samples was measured by plaque assays. Vero cells were cultured on a 12-well plate for plaque formation. The supernatant containing PEDV was added on Vero cells. After 1 h of viral adsorption, the supernatant was removed, and the cells were washed with PBS and overlaid with 0.7% agarose in Dulbecco’s Modified Eagle’s Medium (DMEM) having 2% fetal bovine serum (FBS) and incubated at 37 °C. The plates were fixed with 10% formaldehyde after 72 h infection, and plaques were visualized by staining with Crystal Violet.

### 2.5. Determination of the Viricidal Effect of Salinomycin

PEDV was exposed to solvent DMSO or salinomycin at a concentration of 1 μM for 1 h at 37 °C. Viral infectivity was detected by the TCID50 assay. In further assessments on salinomycin effects on the ability of the viral proliferate, the above-collected viruses were separately inoculated with Vero cells. The viral infectivity was further determined by Western blot.

### 2.6. Western Blot

Vero cells were seeded in 6-well tissue culture plates for 24 h. PEDV infected Vero cells with or without the inhibitor at the indicated times. Then the cells were harvested in 100μL of lysis buffer. Cell lysates were separated by 10% SDS-Page and transferred to a polyvinylidene difluoride (PVDF) membrane. After blocking in 5% skim milk, the membrane was incubated overnight at 4 °C in the presence of specific primary antibodies. Thereafter, the membrane was re-incubated with their corresponding secondary HRP-labeled antibodies at a dilution of 1:4000 for 2 h at room temperature (RT). The membranes were later washed using a TBST buffer and analyzed by film exposure after enhanced chemiluminescence (ECL) reaction.

### 2.7. IFA

Vero cells grown on microscope coverslips in 6-well tissue culture plates were mock-infected or infected with PEDV. Assessment of salinomycin effects on PEDV infection of cells involved pretreating the cells with salinomycin at concentrations of 0.01, 0.1 and 1 μm before infecting them with PEDV. Consequently, cells were fixed in 4% paraformaldehyde for 10 min at room temperature (RT) and permeabilized using 0.2% Triton X−100 in PBS at RT for 10 min. Then, they were blocked using 0.4% bovine serum albumin (BSA) in PBS for 30 min at RT after which they were incubated with PEDV N-specific MAb for 2 h. They were washed 5 times using PBS, re-incubated with a goat anti-mouse secondary antibody conjugated to Alexa Fluor 488 (Invitrogen) for 1 h at RT, then counterstained with DAPI (Sigma). Coverslips were mounted on the microscope glass slides in a mounting buffer. Cell stains were observed using a confocal laser microscope (LSM-710; Zeiss, Oberkochen, Germany) and visualized by CLSM (LSM 710, Zeiss, Oberkochen, Germany).

### 2.8. Cellular ROS Assay

Vero cells in 96-well plates were pretreated with solvent DMSO, salinomycin (1μM) or NAC (5 mM) for 1 h at 37 °C. Then, cells were infected with PEDV in the presence of salinomycin or NAC for 1 h. After washing three times using PBS, fresh medium containing salinomycin or NAC was added. At 24 hpi, the cells were washed three times using PBS and exposed to a reactive oxidative species (ROS) fluorescence indicator H_2_DCFDA (50 μM) at 37 °C for 30 min. Fluorescence was measured in a fluorescence microplate reader.

## 3. Results

### 3.1. Cytotoxicity of Salinomycin on Vero Cells

**Figure 1:**
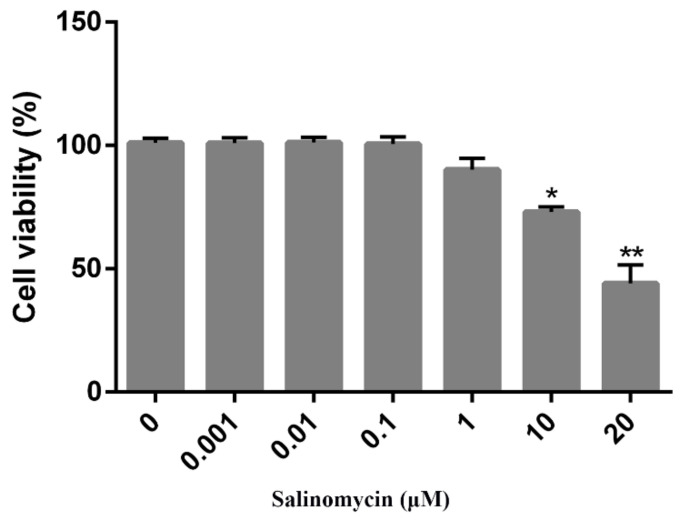
Cytotoxicity of salinomycin in Vero cells. Vero cells treated with salinomycin at indicated concentrations for 24 h at 37 °C or incubated with the culture medium containing DMSO as a control and subjected to CCK8 assay to detect cell viability. Cell viability ranged from 0% to 100%. Data are expressed as three independent experiments. * *p* < 0.05. ** *p* < 0.01.

CCK8 assays were performed to determine the optimal concentration(s) of salinomycin that exhibited minimal cytotoxicity on Vero cells. Salinomycin concentrations ranging from 0.001 to 20 μM were tested. Cytotoxic effects of salinomycin on Vero cells were dose-dependent. At a concentration of 1 μM, there was no toxicity (Figure 1) or alterations in cell morphology when compared to the mock treated cells (data not shown). Consequently, a concentration of less than 1 μM was selected for the subsequent studies.

### 3.2. Antiviral Activity of Salinomycin in Vero Cells

**Figure 2:**
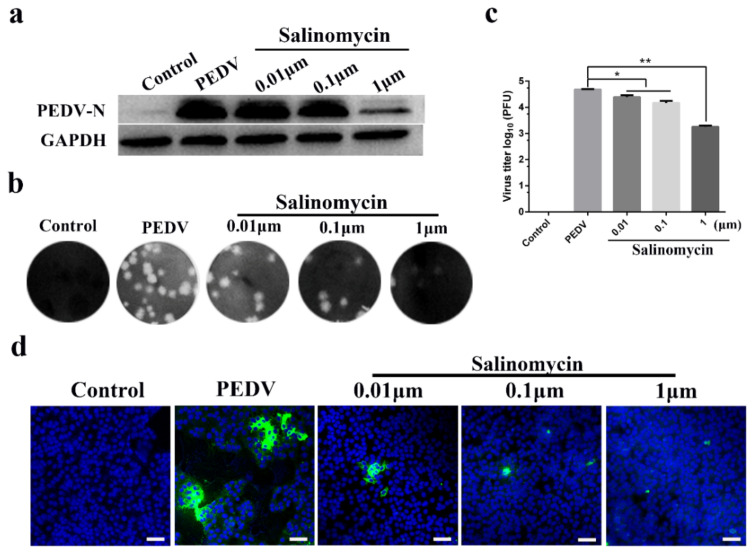
Antiviral activity of salinomycin on porcine epidemic diarrhea virus (PEDV) infection in Vero cells. Vero cells mock pretreated with DMSO or pretreated with salinomycin at concentration of 0.01, 0.1 and 1 μM, respectively. These cells were subjected to PEDV infection for 1 h with or without salinomycin treatment. After extensive washing using PBS, cells were further cultured for 24 h with the medium containing salinomycin or blank medium. Viral yields were then titrated by (**a**) western blot, (**b**) plaque assays and (**d**) indirect immunofluorescence assay (IFA). Green: PEDV N protein; Blue: DAPI; Scale bar, 20 μm. (**c**) Statistical of plaque assays results. The assays were performed in duplicate and data present as means ± SD. * *p* < 0.05, ** *p* < 0.01.

Vero cells were treated with 0.01, 0.1 and 1 μM of salinomycin throughout the PEDV infection cycle, including a pretreatment step for 1 h, during and after each viral inoculation stage. Viral yields were assessed after 24 h using different experimental methods. The N protein of PEDV is composed of 441 amino acids, which is a necessary factor to form the nucleocapsid structure of the virus [15]. Western blot revealed that 1 μM salinomycin decreased in PEDV N protein expression (Figure 2a). Moreover, the antiviral effects of salinomycin at different concentrations were further evaluated through plaque assays (Figure 2b). Accordingly, 0.01, 0.1 and 1 μM salinomycin concentrations revealed a 0.32, 0.57 and 1.45 log reduction in viral yield, respectively (Figure 2c). Viral propagation at 24 h after PEDV infection was then measured using an indirect immunofluorescence assay (IFA; Figure 2d). Treatment of Vero cells with 0.01, 0.1 and 1 μM salinomycin concentrations led to a gradual decrease in PEDV N protein expression in a dose-dependent manner.

### 3.3. Viricidal Effect of Salinomycin on PEDV

**Figure 3:**
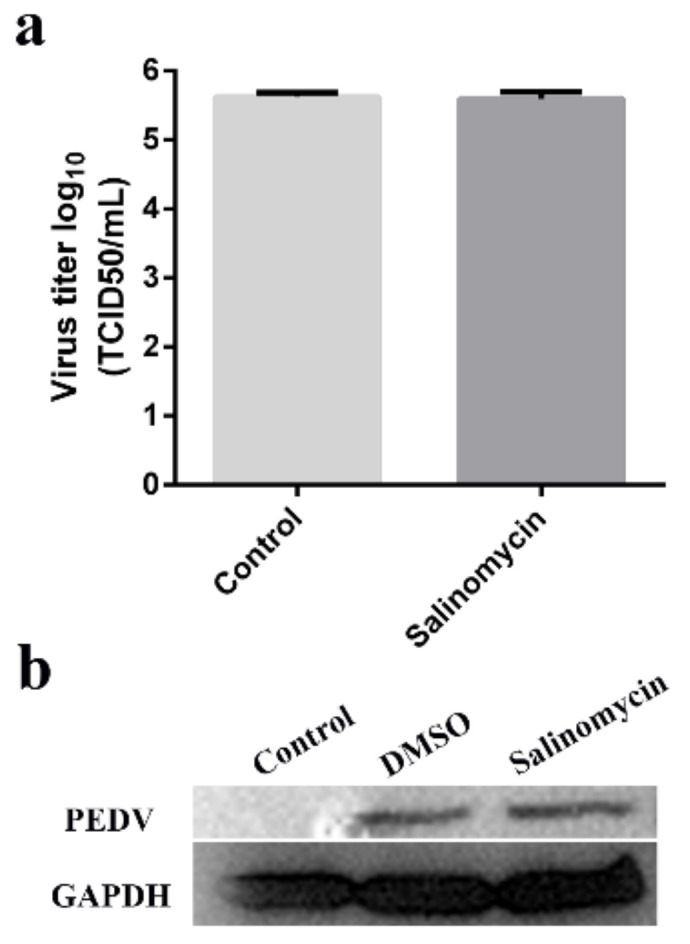
Viricidal effects of salinomycin on PEDV. Viral stocks exposed to salinomycin at 1 μM at 37 °C for 1 h. Subsequently, the viral yields were titrated by the TCID_50_ assays in Vero cells (**a**). The virus propagation in Vero cells after infection for 12 h was checked by western blot (**b**). The assay was performed in duplicate and data presented as the means ± SD.

To determine whether salinomycin directly inactivates PEDV viral particles, the virions were treated with 1 μM salinomycin or with DMSO for 1 h at 37 °C. Subsequently, viruses were titrated through TCID50 assays and western blot. Exposure of PEDV to 1 μM salinomycin did not exhibit any virucidal effect (Figure 3a). Western blot revealed that salinomycin at a concentration of 1 μM did not impair viral propagation (Figure 3b).

### 3.4. Salinomycin Affected PEDV Entry into Vero Cells

**Figure 4:**
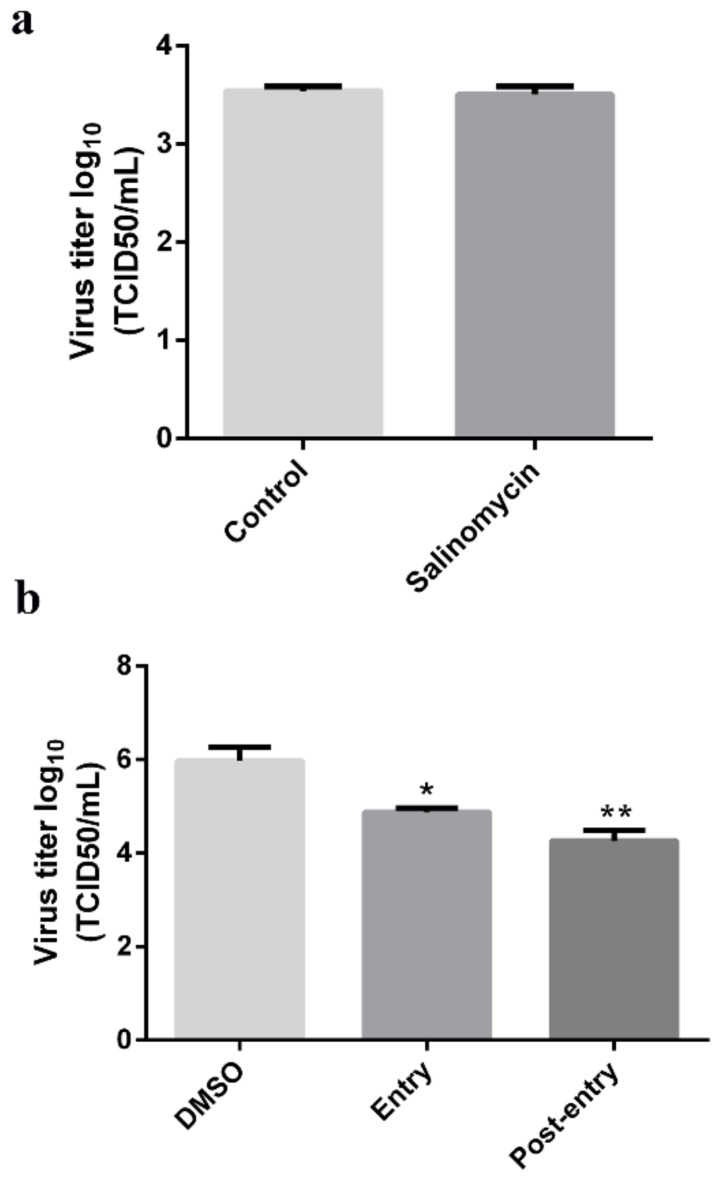
Antiviral effects of salinomycin on different stages of PEDV infectious cycle. Vero cells infected with PEDV were mock-treated with DMSO or treated with salinomycin at different stages of infection. (**a**) Viral titers (log10 TCID50/mL) from cells treated with salinomycin at the viral binding, (**b**) entry and replication stages as calculated by the method of Reed and Muench. The assays were performed in duplicate and data presented as means ± SD. * *p* < 0.05. ** *p* < 0.01.

To establish whether salinomycin exhibits anti-PEDV activity, a time-of-addition analysis was performed to determine where the salinomycin blocks the infection. Then, the efficacy of salinomycin in blocking viral binding was tested by pretreating Vero cells with salinomycin then exposing them to viral particles along with DMSO or salinomycin for 1 h at 4 °C. After extensive washing, cells were subjected to two rounds of freezing-thawing before cell-attached viruses were titrated through the TCID50 assay. No significant inhibitory effect on viral binding was observed at 1 μM salinomycin, indicating that salinomycin did not affect PEDV attachment to Vero cells (Figure 4a). Interestingly, when administered at the entry and post-entry stage, 1 μM of salinomycin significantly inhibited viral reproduction (Figure 4b). Taken together, these results imply that salinomycin inhibited PEDV entry into cells and PEDV replication in infected cells.

### 3.5. Salinomycin Inhibited PEDV Infection Stimulated MAPK Signaling

**Figure 5:**
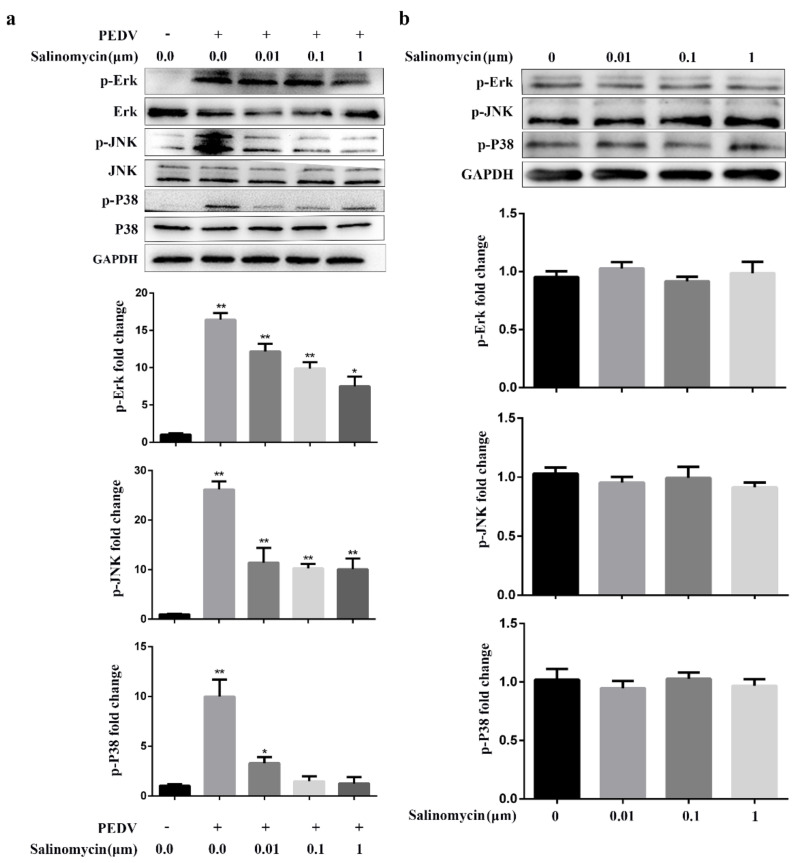
Effect of salinomycin on MAPK signaling in response to PEDV infection. (**a**) Serum starved Vero cells pretreated with salinomycin at the indicated concentrations for 1 h, respectively, then infected with PEDV in the presence of salinomycin for 24 hpi. The cell lysate was prepared and subjected to western blotting analysis. (**b**) Serum starved Vero cells were exposed to salinomycin at the indicated concentrations for 24 h. Cell lysate was prepared and subjected to western blotting analysis. The band intensity was analyzed with software image J. These results represented three independent experiments and data presented as means ± SD. * *p* < 0.05. ** *p* < 0.01.

The JNK, Erk and p38 MAPK signaling activation is required for PEDV replication. In addition, previous studies have shown that salinomycin partly exerts its pharmacological roles in human prostate cancer cells through the MAPK signaling pathway, suggesting that salinomycin may inhibit PEDV infection through MAPK signaling [16]. Therefore, the effects of salinomycin on MAPK signaling in response to PEDV infection were evaluated. PEDV induced activated Erk, JNK and p38 MAPK, attenuated by salinomycin in a dose-dependent manner. However, these inhibitory outcomes had no drastic effects on total steady-state protein levels of Erk, JNK and p38 MAPK following PEDV infection (Figure 5a). Furthermore, as shown in Figure 5b, salinomycin did not exert a significant effect on the levels of phosphorylated Erk, JNK and p38 MAPK in mock-infected cells. Therefore, the inhibitory effects of salinomycin on PEDV-infection was partially dependent on the activation of Erk, JNK and p38 MAPK signaling.

### 3.6. PEDV Induced ROS Generation Was Not Decreased by Salinomycin

**Figure 6:**
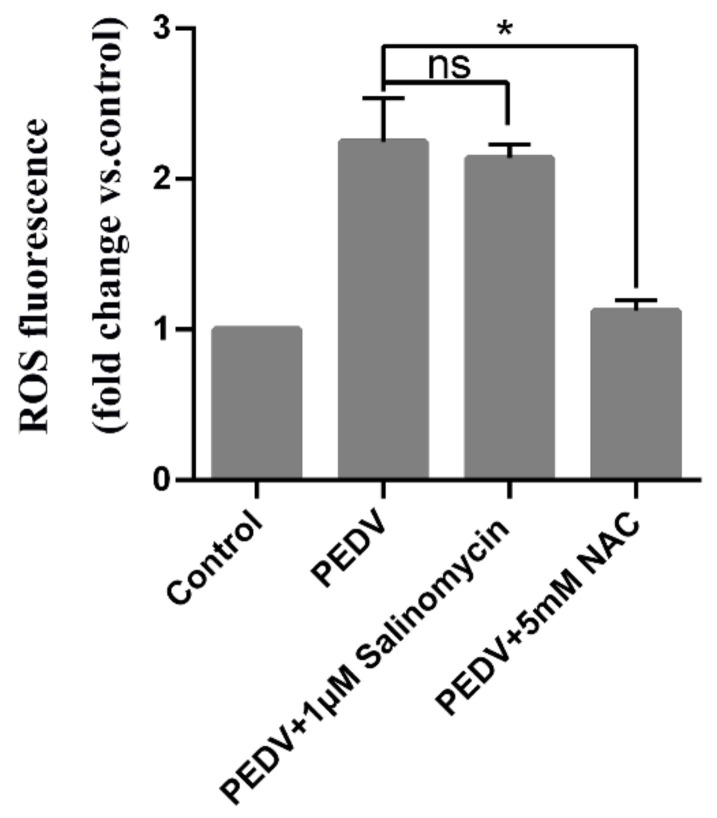
Inhibition of PEDV replication by salinomycin is independent of reactive oxidative species (ROS) generation. Vero cells subjected to pretreatment with NAC (5 mM) or salinomycin(1 μM) for 1 h, were infected with PEDV along with NAC or salinomycin. At 24 hpi cellular ROS were detected using H_2_DCFDA (5 μM, 30 min). Values represent three independent experiments. Significant differences compared to untreated infected cells are indicated by *, (* *p* < 0.05), ns, no significance.

Studies have shown that PEDV infection enhances ROS production. Therefore, we further assessed the potential of salinomycin to inhibit PEDV-induced ROS production. Figure 6 shows that 24 h post-infection, PEDV infection resulted in elevated intracellular ROS levels, which were strongly decreased by NAC but not by salinomycin treatments. Therefore, salinomycin suppresses PEDV replication through a pathway that is independent of viral-induced ROS.

## 4. Discussion

Vaccination and effective therapies are fundamental strategies for controlling and eradicating viral infections [17]. There are several veterinary coronavirus vaccines available, with varied efficacies. The vaccine for infectious bronchitis virus (IBV) in chicken has been shown to be effective [18]. However, effective vaccines or specific treatments for PEDV have not yet been developed despite several compounds demonstrating promising antiviral activities in PEDV-infected Vero cells [19,20,21,22,23,24,25]. In this study, for the first time, we demonstrated the inhibitory effects of salinomycin on PEDV infection.

Salinomycin has been widely used as an anticoccidiosis agent, and has been shown to enhance the sensitivity of cancer cells to radiation and chemotherapeutic treatments [26,27,28]. Moreover, salinomycin has been reported to inhibit influenza virus infection [13]. Both PEDV and influenza viruses are RNA viruses that share several biological properties, implying that salinomycin might inhibit other RNA viruses. The PEDV life cycle consists of four stages: attachment, entry, replication and release [15]. In this study, salinomycin was found to significantly inhibit the entry and replication of PEDV in Vero cells in a dose dependent manner. However, it did not affect PEDV binding to Vero cells, implying that it did not directly inhibit viral attachment to cell receptors. Furthermore, salinomycin did not directly interact with or inactivate PEDV particles, as pre-exposure of the virus to salinomycin did not alter the infectivity of PEDV particles.

Various viruses modulate inflammatory responses by regulating the MAPK pathway to facilitate their replication, regulate cell proliferation and inhibit cell apoptosis or induce cytokine production [29,30,31]. In addition, ERK1/2, p38MAPK and JNK signaling pathways in PEDV-infected cells are activated to facilitate viral replication [32,33,34]. In this study, activations of Erk1/2, JNK and p38MAPK signaling by PEDV infection were significantly ameliorated by salinomycin treatment, especially at the concentration of 1 μM, suggesting that salinomycin inhibited PEDV replication through interfering with the MAPK pathway activation. This is consistent with the findings of other studies. For example, salinomycin was shown to inhibit tumor cell proliferation through the MAPK/ ERK1/2 pathway [16]. It has been reported that PEDV can induce reactive oxygen species (ROS) production. We confirmed that salinomycin cannot reverse PEDV infection-induced ROS accumulation. This is similar to the reports that salinomycin overcame radioresistance in nasopharyngeal carcinoma cells by reducing Nrf2 level and promoting ROS generation [35]. Salinomycin not only cannot inhibit ROS production, but can promote ROS production. Therefore, inhibition of PEDV replication by salinomycin is not associated with ROS production and the mechanisms through which salinomycin inhibits PEDV replication should be further evaluated in vitro.

In conclusion, salinomycin is a potent PEDV inhibitor that can be used to treat PEDV infections. More studies should be performed to evaluate the antiviral effects as well as the mechanisms of salinomycin in preventing various PEDV-mediated injuries and infections in pathological situations in vivo. Furthermore, studies should evaluate the potential antiviral properties of salinomycin against other coronaviruses, including severe acute respiratory syndrome coronavirus 2 (SARS-CoV-2), severe acute respiratory syndrome coronavirus (SARS-CoV) and middle east respiratory syndrome coronavirus (MERS-CoV).

## Data Availability

Not applicable.

## Abbreviations

PEDV	porcine epidemic diarrhea virus
PED	Porcine epidemic diarrhea
ROS	reactive oxygen species
IBV	infectious bronchitis virus
SARS-CoV-2	severe acute respiratory syndrome coronavirus 2
SARS-CoV	severe acute respiratory syndrome coronavirus
MERS-CoV	middle east respiratory syndrome coronavirus
